# Linagliptin Protects Human SH-SY5Y Neuroblastoma Cells against Amyloid-β Cytotoxicity via the Activation of Wnt1 and Suppression of IL-6 Release

**DOI:** 10.52547/ibj.25.5.343

**Published:** 2020-10-07

**Authors:** Mohsen Sedighi, Tourandokht Baluchnejadmojarad, Mehrdad Roghani

**Affiliations:** 1 Neuroscience Research Center, Iran University of Medical Sciences, Tehran, Iran;; 2 Department of Physiology, School of Medicine, Iran University of Medical Sciences, Tehran, Iran;; 3 Neurophysiology Research Center, Shahed University, Tehran, Iran

**Keywords:** Alzheimer disease, Interleukin-6, Linagliptin, Wnt1 protein

## Abstract

**Background:**

Alzheimer’s disease is one of the neurodegenerative disorders typified by the aggregate of Aβ and phosphorylated tau protein. Oxidative stress and neuroinflammation, because of Aβ peptides, are strongly involved in the pathophysiology of AD. Linagliptin shows neuroprotective properties against AD pathological processes through alleviation of neural inflammation and AMPK activation.

**Methods:**

We assessed the benefits of linagliptin pretreatment (at 10, 20, and 50 nM concentrations), against Aβ1-42 toxicity (20 μM) in SH-SY5Y cells. The concentrations of secreted cytokines, such as TNF-α, IL-6, and IL-1β, and signaling proteins, including pCREB, Wnt1, and PKCε, were quantified by ELISA.

**Results:**

We observed that Aβ led to cellular inflammation, which was assessed by measuring inflammatory cytokines (TNF-α, IL-1β, and IL-6). Moreover, Aβ1-42 treatment impaired pCREB, PKCε, and Wnt1 signaling in human SH-SY5Y neuroblastoma cells. Addition of Linagliptin significantly reduced IL-6 levels in the lysates of cells, treated with Aβ1-42. Furthermore, linagliptin prevented the downregulation of Wnt1 in Aβ1-42-treated cells exposed.

**Conclusion:**

The current findings reveal that linagliptin alleviates Aβ1-42-induced inflammation in SH-SY5Y cells, probably through the suppression of IL-6 release, and some of its benefits are mediated through the activation of the Wnt1 signaling pathway.

## INTRODUCTION

Alzheimer’s disease, the most usual neurodegenerative disorder, is typified by the accumulation of extracellular Aβ plaques, intracellular hyperphosphorylated tau protein, and neurofibrillary tangles^[^^1^^]^. While the accurate mechanisms of neurodegeneration are not yet fully understood, recent report suggest that the development of neuroinflammation and oxidative stress process strongly contribute to the pathogenesis of neurodegenerative diseases, as well as AD^[^^2^^]^. Numerous investigations have shown an upregulation of inflammatory agents and activated glial cell in the brain of AD patients and AD transgenic animal models^[^^3^^]^. IL-6, IL-1β, and TNF-α are strong mediators of neural inflammation, which is responsible for the pathogenesis of AD^[^^4^^]^. Release of cytokines due to inflammation recruits circulating monocytes and lymphocytes to promote neural inflammation in the CNS^[^^5^^]^. 

Recent surveys have indicated that GLP-1 analogues ameliorate neurodegeneration in AD. This incretin hormone can cross the blood-brain barrier and plays a mediatory role in the CNS. Nevertheless, GLP-1 is rapidly inactivated by DPP-4, a serine peptidase in the bloodstream, leading to decreased GLP-1 half-life. It has been documented that DPP-4 inhibitors such as linagliptin show neuroprotection by the increase of GLP-1 activity in the circulation. It has been reported that linagliptin improves Aβ-induced cytotoxicity via the induction of AMPK signaling pathway and the Sirt1-elicited antioxidant pathways such as superoxide dismutase in neuronal cells.

Wnt signaling cascade is involved in the regulation of synaptic transmission and plasticity in the brain^[^^6^^]^. Research reports have indicated that Wnt signaling impairment plays a pivotal role in AD pathogenesis^[^^7^^]^. Interestingly, Wnt signaling actively contributes to the Aβ formation and impairment of Wnt signaling pitches in the appearance of tau phosphorylation^[^^8^^,^^9^^]^. In addition, Wnt1 exhibits neuroprotective effects against amyloid plaque formation and phosphorylation of tau protein. Therefore, impairment of the Wnt/β-catenin pathway could act in AD development^[^^10^^,^^11^^]^. 

PKC is a family member of isoenzymes of serine/threonine protein kinases acting distinctly in the regulation of cellular signal transduction^[^^12^^]^. It has been reported that PKCε significantly involves in the regulation of APP metabolism and regulation of diverse functions in neuronal cells, including the modulation of gene expression^[^^13^^]^. Moreover, CREB is crucial for neuronal survival and function^[^^14^^]^. It is well documented that CREB-mediated gene expression is damaged in the AD brain, and the level of phosphorylated CREB declines in the hippocampal neurons of PS1/APP double mutant transgenic mice^[^^15^^]^. The human SH-SY5Y cell line has been widely used in neuroscience research, particularly in the generation of the cellular model of neurodegenerative diseases^[^^16^^-^^18^^]^. The current investigation was aimed to explore the protective effects of linagliptin as a DPP-4 inhibitor on the Aβ-induced cytotoxicity in SH-SY5Y cells through the evaluation of Wnt1, PKCε, and CREB signaling, as well as inflammatory cytokines.

## MATERIALS AND METHODS

**Materials**

Human neuroblastoma SH-SY5Y cells (Pasteur Institute of Iran, Teran) and MTT assay (BIO-IDEA, Iran) were used in this study. Chemicals, such as Aβ1-42 and pure linagliptin were acquired from R&D Systems (USA) and Cayman Chemical (USA), respectively. Antibodies purchased for ELISA were as follow: IL-6, IL-1β, Wnt1, PKCε (MyBioSource, USA), TNF-α (Sigma-Aldrich, USA), and pCREB (R&D Systems).

**Preparation of Aβ and linagliptin**

Recombinant Aβ1-42 was prepared according to a previously described method^[^^19^^]^. Briefly, Aβ1-42 peptide was solubilized in 10% DMSO to acquire a 2-μM solution, which was consisted of fibrillar and monomer forms of Aβ1-42. For the preparation of linagliptin, pure linagliptin powder was dissolved in DMSO for 24 h to obtain 10, 20, and 50 μM solutions. The range of concentrations for linagliptin was opted based on an earlier investigation on the protective effect of linagliptin against Aβ-induced cytotoxicity and insulin signaling impairment in SK-N-MC human neuronal cells^[^^20^^]^. All preparations were incubated at 4 °C for 24 h and stored at -20 °C. 

**Cell culture and viability assay**

DMEM/F12 supplemented with penicillin 100 U/ml, 10% FBS, and streptomycin 100 μg/ml was used at conditions of 37 °C, 5% CO_2 _and 95% air to culture SH-SY5Y cells. The cells were seeded onto 96-welled plates to reach a density of 5000 cell/well and incubated for 24 h. Pretreatment of cells was carried out with various doses of linagliptin for 24 h and followed by the exposure of cells to Aβ1-42 overnight. Experimental groups included SH-SY5Y cells without treatment (control group), linagliptin groups (SH-SY5Y cells pretreated with 10, 20, and 50 μM of linagliptin), Aβ group (SH-SY5Y cells treated with 20 μM of Aβ), Aβ + linagliptin groups (SH-SY5Y cells treated with 20 μM of Aβ and 10, 20, and 50 μM linagliptin). To conduct the viability assay, we added MTT solution to the medium following the linagliptin pretreatment and Aβ challenge. After 4 h, the reaction product was solubilized by DMSO. Culture plates containing SH-SY5Y cells were placed overnight (37 °C), and a microplate reader was applied to measure absorbance at 570 nm. The results were compared with the untreated cells, as the control group. 

**ELISA technique**

Treated SH-SY5Y cells were incubated with hypotonic lysis buffer after harvesting. Cells were centrifuged at 10,000 ×g in a centrifuge at 4 °C to obtain the cell lysate. The levels of secreted cytokines (IL-6, IL-1β, and TNF-α) and signaling proteins (pCREB, Wnt1, and PKCε) in the cell lysate were quantified using ELISA.

**Statistical analysis**

SPSS package (version 22, Chicago, IL, USA) was applied for data analysis, and one-way ANOVA followed by Turkey’s post hoc test was utilized for the comparison of experimental groups. Outcomes were presented as mean ± SEM, and a *p *value <0.05 was considered statistically significant in all experiments.

**Fig. 1:**
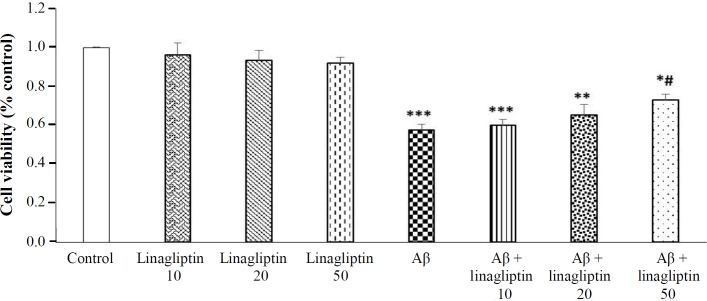
The effect of Aβ 1-42 (20 μM) and linagliptin (10, 20, and 50 nM) on the viability of human SH-SY5Y neuroblastoma cells. Cells were incubated with linagliptin for 24 h, and then Aβ was added for an additional 24 h. ^*** ^*p* < 0.001, ^**^p < 0.01, ^*^p < 0.05 (as compared to control); ^#^*p* < 0.05 (as compared to Aβ 1-42)

## RESULTS

**Effects of Aβ1-42 and linagliptin on the viability of SH-SY5Y cells**

Pretreatment of SH-SY5Y cells with linagliptin (10, 20, and 50 μM) was accomplished for 24 h, and the cell viability was assessed by the MTT test in order to explore whether linagliptin induces cytotoxicity in these cells. As represented in Figure 1, cell viability in the treated cells with linagliptin (10, 20, and 50 μM) did not show any significant change. Therefore, we selected 50 μM of linagliptin in later experiments. Moreover, the toxicity of Aβ1-42 on cell viability was assessed via incubating SH-SY5Y cell with Aβ1-42 (20 μ M). Exposure of cells to Aβ1-42 significantly reduced the cell viability of cultured SH-SY5Y cells (*p* < 0.001). Furthermore, pretreatment of cells with 50 μM of linagliptin significantly prevented the decrease of viability in cells treated with Aβ1-42. 

**Effect of Aβ1-42 and linagliptin on inflammatory cytokines on SH-SY5Y cells**

**Fig. 2:**
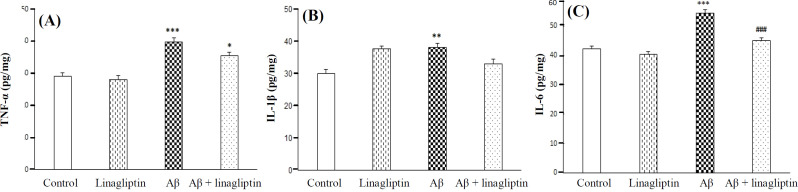
The effect of Aβ 1-42 (20 μM) and linagliptin (50 nM) on TNF-α, IL-1β, and IL-6 levels in human SH-SY5Y neuroblastoma cells. Cells were incubated with linagliptin for 24 h, and then Aβ was added for an additional 24 h. ^***^p < 0.001, ^**^p < 0.01 (as compared to control); ^###^*p* < 0.001 (as compared to Aβ 1-42)

To investigate the effects of Aβ1-42 and linagliptin on inflammatory biomarkers, we measured the concentrations of TNF-α, IL-1β, and IL-6 in cell lysate. The concentrations of TNF-α (*p* < 0.001), IL-1β (*p* < 0.01), and IL-6 (*p* < 0.001) in treated cells were risen significantly after exposure to Aβ1-42. Furthermore, the pretreatment of cells with linagliptin could significantly decrease the levels of IL-6 in Aβ1-42 exposed cells (*p* < 0.001; Fig 2). 

**Effects of Aβ 1-42 and linagliptin on pCREB, PKCε, and Wnt1 levels on SH-SY5Y cells**

**Fig. 3:**
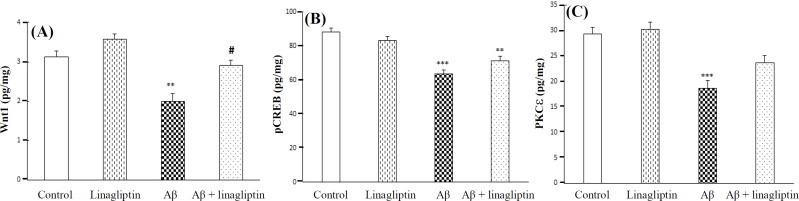
The effect of Aβ 1-42 (20 μM) and linagliptin (50 nM) on Wnt1, pCREB, and PKCε in human SH-SY5Y neuroblastoma cells. Cells were incubated with linagliptin for 24 h, and then Aβ was added for an additional 24 h. ^***^p < 0.001, ^**^p < 0.01 (as compared to control); ^#^*p* < 0.05 (as compared to Aβ 1-42)

The lysate levels of Wnt1 (*p* < 0.001), pCREB (*p* < 0.001), and PKCε (*p* < 0.001) noticeably declined in cells treated with Aβ 1-42, as shown in Figure 3. Furthermore, the pretreatment of cells with linagliptin prevented the down-regulation of the lysate level of Wnt1, in cells treated with Aβ 1-42. 

## DISCUSSION

AD is a prevalent form of neurodegenerative diseases characterized by progressive memory loss and a slow decrease in cognitive function. Normally,

The accumulation of extracellular senile plaques containing Aβ, neurofibrillary tangles, and abnormal phosphorylated tau protein are involved in the AD pathogenesis, especially in the hippocampus and cortex^[^^20^^,^^21^^]^. 

The present study was designed to test the potential effect of linagliptin on Aβ-induced cytotoxicity. Our results revealed that the exposure of SH-SY5Y cells to Aβ1-42 exerts cellular cytotoxicity, which leads to an inflammatory response and induced release of cytokines such as TNF-α, IL-6, and IL-1β. Our findings implicate the role of the inflammatory process in AD pathophysiology^[^^3^^,^^22^^,^^23^^]^. It has been well defined that cytokines released from activated astrocytes and microglia are the main effectors of neural inflammation signals that can affect cognitive function and memory in AD^[^^4^^,^^24^^,^^25^^]^. Moreover, the cytotoxic effect of Aβ1-42 is mediated through the inhibition of Wnt1, pCREB, and PKCε signaling pathways^[^^26^^-^^28^^]^.

Recent investigation has reported that incretins may be a feasible choice for AD treatment^[^^29^^]^. In addition to the increase of GLP-1 levels, one study has shown that linagliptin, as a DPP-4 inhibitor, exhibits a neuroprotective effect on Aβ-induced neurotoxicity associated with AD^[^^27^^]^. Our prior investigation has discovered that the treatment of peripheral mononuclear blood cells with linagliptin decreases the cellular levels of IL-1β in healthy individuals and TNF-α in AD patients^[^^11^^]^. In the current research, findings indicated that linagliptin pretreatment attenuates increased IL-6 in cells cultured with Aβ1-42. Typically, IL-6 is the most inflammatory marker released by activated microglia and astrocytes in different regions of the brain. It is also capable of stimulating microglia and astrocytes to produce a cascade of proinflammatory cytokines^[^^30^^]^. Former studies have established that IL-6 involves in the APP processing and production in primary rat cortical neurons^[^^31^^,^^32^^]^. Nakamura *et al.*^[^^33^^]^ have reported that linagliptin remarkably reduces p65 expression, p38 MAPK phosphorylation, and IL-6 production in the endothelial cells of the umbilical vein, which were treated with lipopolysaccharides. Furthermore, research findings have provided evidence for this point that linagliptin ameliorates Aβ-induced cytotoxicity via the stimulation of AMPK and the Sirt1-elicited antioxidant pathways^[^^20^^]^.

The Wnt signaling cascade regulates plasticity and synaptic transmission in the nervous system and the link between the Wnt signaling pathway and AD pathogenesis has been well documented^[^^34^^,^^35^^]^. Accordingly, research findings have proposed that Wnt signaling deficiency is an important contributing factor in the formation of Aβ and the etiology of AD^[^^36^^]^. Tapia-Rojas and Nibaldo^[^^6^^]^ have reported that the suppression of the Wnt signaling leads to a rise in Aβ 42 levels and the Aβ42/Aβ40 ratio, which is in favor of Aβ oligomerization *in vitro*. In this study, exposure of neuroblastoma cells to Aβ1-42 significantly decreased Wnt1 level, which is consistent with an investigation^[^^26^^]^. The activation of canonical Wnt signaling resulted in the protective effect of Trolox and vitamin C against Aβ-induced cytotoxicity in hippocampal cultured neurons. A recent discovery has shown that the treatment of isolated hippocampal neurons with some antioxidants protects neurons against Aβ neurotoxicity by a mechanism involving the activation of Wnt signaling and control of oxidative stress^[^^37^^]^. Interestingly, some antidiabetic drugs, including DPP-4 inhibitors, have demonstrated a beneficial effect in the CNS of experimental models of AD^[^^20^^,^^38^^-^^40^^]^. We found the increased expression of Wnt1 level in the lysate of SH-SY5Y cells that were pretreated with linagliptin before Aβ challenge. Our data support this hypothesis that the modulation of Wnt1 signaling might play an important role in the protection against Aβ_-_induced cytotoxicity^[^^37^^,^^41^^]^. Moreover, we add this evidence that DPP-4 inhibitor linagliptin has ability to protect SH-SY5Y cells against Aβ challenge, possibly via the activation of Wnt1 signaling and control of oxidative stress.

Our findings in this study exhibit that linagliptin ameliorates Aβ-induced cytotoxicity in human neuroblastoma cells, and a part of this protective effect is mediated through the activation of Wnt1 signaling and control of cellular inflammation. Hence, linagliptin may be a promising therapeutic candidate to treat AD, but more investigations are needed to support this hypothesis. 
